# Hybrid artificial neural network and structural equation modelling techniques: a survey

**DOI:** 10.1007/s40747-021-00503-w

**Published:** 2021-08-28

**Authors:** A. S. Albahri, Alhamzah Alnoor, A. A. Zaidan, O. S. Albahri, Hamsa Hameed, B. B. Zaidan, S. S. Peh, A. B. Zain, S. B. Siraj, A. H. B. Masnan, A. A. Yass

**Affiliations:** 1 Faculty of Human Development, Sultan Idris University of Education (UPSI), Tanjung Malim, Malaysia; 2 Department of Computing, Sultan Idris University of Education (UPSI), Tanjong Malim, Malaysia; 3 Informatics Institute for Postgraduate, Studies (IIPS), Iraqi Commission for Computers and Informatics, Baghdad, Iraq; 4 School of Management, Management Technical College, 11800 Gelugor, Pulau Pinang Malaysia; 5 Southern Technical Univeristy, Management Technical College, Basrah, Iraq; 6 IT Infra Expert Company, Earthlink, Main ISP, Baghdad, Iraq; 7 National Yunlin University of Science and Technology, Future Technology Research Center, Yunlin, Taiwan, ROC

**Keywords:** Structural equation modelling, Artificial neural network, Therapy, Autistic children

## Abstract

Topical treatments with structural equation modelling (SEM) and an artificial neural network (ANN), including a wide range of concepts, benefits, challenges and anxieties, have emerged in various fields and are becoming increasingly important. Although SEM can determine relationships amongst unobserved constructs (i.e. independent, mediator, moderator, control and dependent variables), it is insufficient for providing non-compensatory relationships amongst constructs. In contrast with previous studies, a newly proposed methodology that involves a dual-stage analysis of SEM and ANN was performed to provide linear and non-compensatory relationships amongst constructs. Consequently, numerous distinct types of studies in diverse sectors have conducted hybrid SEM–ANN analysis. Accordingly, the current work supplements the academic literature with a systematic review that includes all major SEM–ANN techniques used in 11 industries published in the past 6 years. This study presents a state-of-the-art SEM–ANN classification taxonomy based on industries and compares the effort in various domains to that classification. To achieve this objective, we examined the Web of Science, ScienceDirect, Scopus and IEEE *Xplore*^®^ databases to retrieve 239 articles from 2016 to 2021. The obtained articles were filtered on the basis of inclusion criteria, and 60 studies were selected and classified under 11 categories. This multi-field systematic study uncovered new research possibilities, motivations, challenges, limitations and recommendations that must be addressed for the synergistic integration of multidisciplinary studies. It contributed two points of potential future work resulting from the developed taxonomy. First, the importance of the determinants of play, musical and art therapy adoption amongst autistic children within the healthcare sector is the most important consideration for future investigations. In this context, the second potential future work can use SEM–ANN to determine the barriers to adopting sensing-enhanced therapy amongst autistic children to satisfy the recommendations provided by the healthcare sector. The analysis indicates that the manufacturing and technology sectors have conducted the most number of investigations, whereas the construction and small- and medium-sized enterprise sectors have conducted the least. This study will provide a helpful reference to academics and practitioners by providing guidance and insightful knowledge for future studies.

## Introduction

Applied business research was presented in accordance with Wynne W. Chin in the late 1990s [1]. Hence, the structural equation modelling (SEM) technique has evolved rapidly as an extremely broad, flexible and important statistical tool [2]. SEM is a second-generation multivariate analysis technique that is easy to use and provides high-quality statistical analyses [3]. The authors of [4] identified two types of SEM: covariance-based SEM (CB-SEM) and partial least squares SEM (PLS-SEM). CB-SEM is adopted to confirm a well-established theory or explanation, whilst PLS-SEM is used in exploratory and confirmatory research. PLS-SEM achieves higher predictive accuracy in causal explanations than CB-SEM [5]. PLS-SEM is becoming a popular method in social research [6], and many authors have contributed to address hard problems that cannot be solved using CB-SEM [7]. SmartPLS is one of the software packages that deal with PLS-SEM when analysing conceptual frameworks with one or more dependent variables [8].

Moreover, PLS-SEM is a widely used method in a variety of fields because it is suitable for developing and testing hypotheses and explaining causal relationships [9]. In addition, PLS-SEM exhibits predictive advantages, such as *R*^2^ (coefficient of determination) and *Q*^2^ (predictive relevance) values [10]. The latter is the primary justification for adopting PLS-SEM in the previous literature. PLS-SEM exhibits high potential in solving industrial and academic issues [11]; hence, a new research direction is the prediction of important factors using a dual-stage model of SEM and an artificial neural network (ANN) because SEM is unable to explore nonlinear relationships amongst constructs [12–15]. The purpose of merging SEM with ANN is to explain nonlinear and non-compensatory relationship amongst constraints [16]. In particular, ANN depends on the black box process of the algorithm, which is the basis for prediction [17]. ANN can capture linear and nonlinear correlations amongst variables, and thus, provide more accurate results. This feature contributes to each variable in the model, overcoming the weaknesses of multiple regression analysis, SEM and logistic analysis. However, ANN analysis is unsuitable for testing hypotheses because it depends on the black box process of the algorithm [18].

Consequently, ANN is defined as a parallel circulating processor consisting of processing units with a neural tendency to store experimental information and make it available for usage [19]. Moreover, ANN provides the rank and validates the significant predictors of a model [20]. Several studies have used systematic literature reviews to apply PLS-SEM in the marketing and hospitality sectors [21–23]. Although such reviews are crucial for ensuring research practices, they do not consider studies that used techniques combined with PLS-SEM (e.g. ANN) [1]. Thus, whether authors have correctly applied PLS-SEM with ANN to their research problems and whether they are fully utilizing the potential of deep learning-based two-staged hybrid SEM–ANN analysis cannot be determined [24]. Consequently, the literature that uses SEM with ANN has expanded, but the various forms of study cases that connect SEM with ANN remain unknown and should be investigated further. The present systematic analysis aims to provide useful insights into the contexts of SEM and ANN and assist authors in identifying current options and gaps in this line of study. Moreover, the current work aims to determine the highlights of authors’ effort in response to new statistical analysis directions (i.e. dual-stage analysis of SEM–ANN), map the study context into a coherent taxonomy and identify the features that refer to this developing line of research in SEM and ANN.

## Methods

The present research was conducted to comply with the criteria established in the Preferred Reporting Items for Systematic Reviews and Meta Analyses (PRISMA) [25–29]. PRISMA recommends not relying on a single database search for literature when doing a systematic review because no single database is likely to include all relevant references. Consequently, considerable research is nearly mandatory [30–35]. In accordance with previous studies [36–40], an accurate and thorough survey may be performed on several databases to cover numerous articles.

### Search strategy

**Fig. 1:**
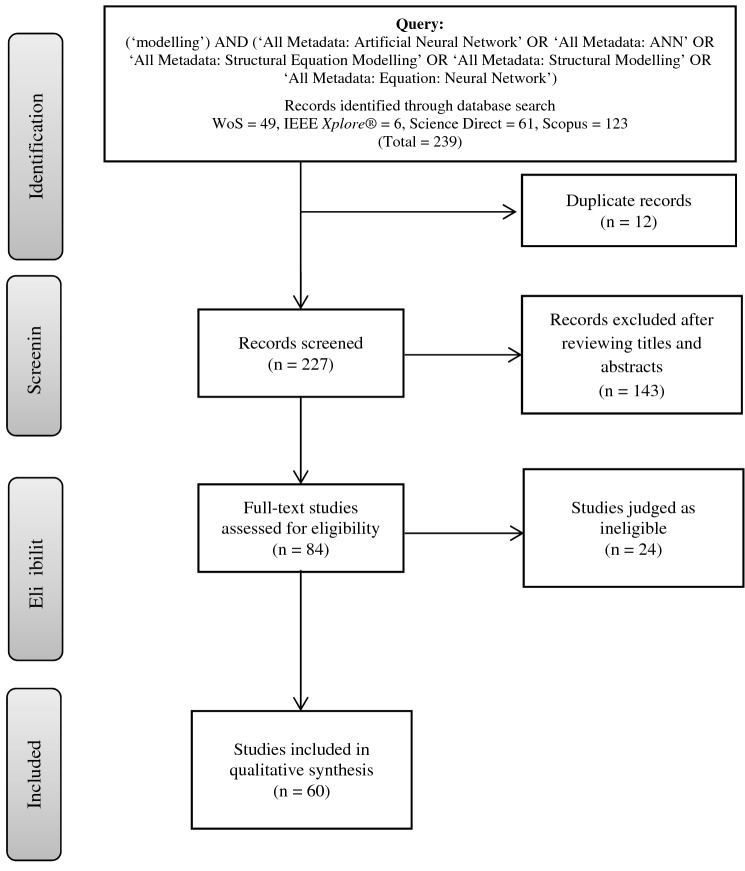
Flowchart of the processes of classifying, screening and including relevant studies

A wide-ranging literature search was conducted in IEEE *Xplore*^®^, ScienceDirect, Web of Science (WoS) and Scopus for English language citations published over 6 years (2016–2021). These online databases were selected because they include a significant number of pertinent papers that span scientific, technological and clinical aspects in their interdisciplinary research [41–45]. Different keywords were used to perform the search [46–50]. Primary keywords include ‘structural equation modelling’ and ‘artificial neural network’, as described in Fig. 1.

### Inclusion and exclusion criteria

The following criteria were used to identify relevant research.

1. The study is a published journal article or conference paper written in English.
2. The included articles should apply the SEM–ANN model regardless of the focus of the study.

Papers published in a language other than English were excluded from the study. Articles that did not satisfy the inclusion criteria for this research, such as those that did not utilise the SEM–ANN model or those that used a different analysis method, were also rejected. Moreover, articles that were not related to SEM–ANN, such as PLS-SEM studies, or those about other topics that were irrelevant to SEM–ANN were excluded.

### Study selection

Consequently, duplicates were eliminated. Three researchers conducted an in-depth search for papers that fit the criteria, examining the titles and abstracts. Finally, each researcher evaluated all the relevant articles that he/she could find. Subsequently, additional researchers examined the entire process, including the extraction of the generated data tables. To overcome possible consultation disagreements, a senior researcher (i.e. the corresponding author) who already read and examined all the included articles served as the mediator and inspector to ascertain and ensure quality.

### Data collection and classification

Two filtering procedures were performed to find relevant papers in the area of SEM–ANN research. The first filter, which searches for articles on the basis of their titles and abstracts, helps eliminate irrelevant articles. The second filter relies on a thorough reading of an article to identify any remaining irrelevant papers. The researchers tracked the entire data collection process, ensuring that the resulting systematic review was as consistent and rigorous as possible. Additional data were collected from the academic literature and organised into various survey article lists, source indexes, summary tables, description tables, purposes, review sources, audience and used datasets. The categories of the studies mentioned below will assist researchers and developers in thinking critically about the problems that they will investigate in the future.

## Search results and statistical information of articles

**Fig. 2:**
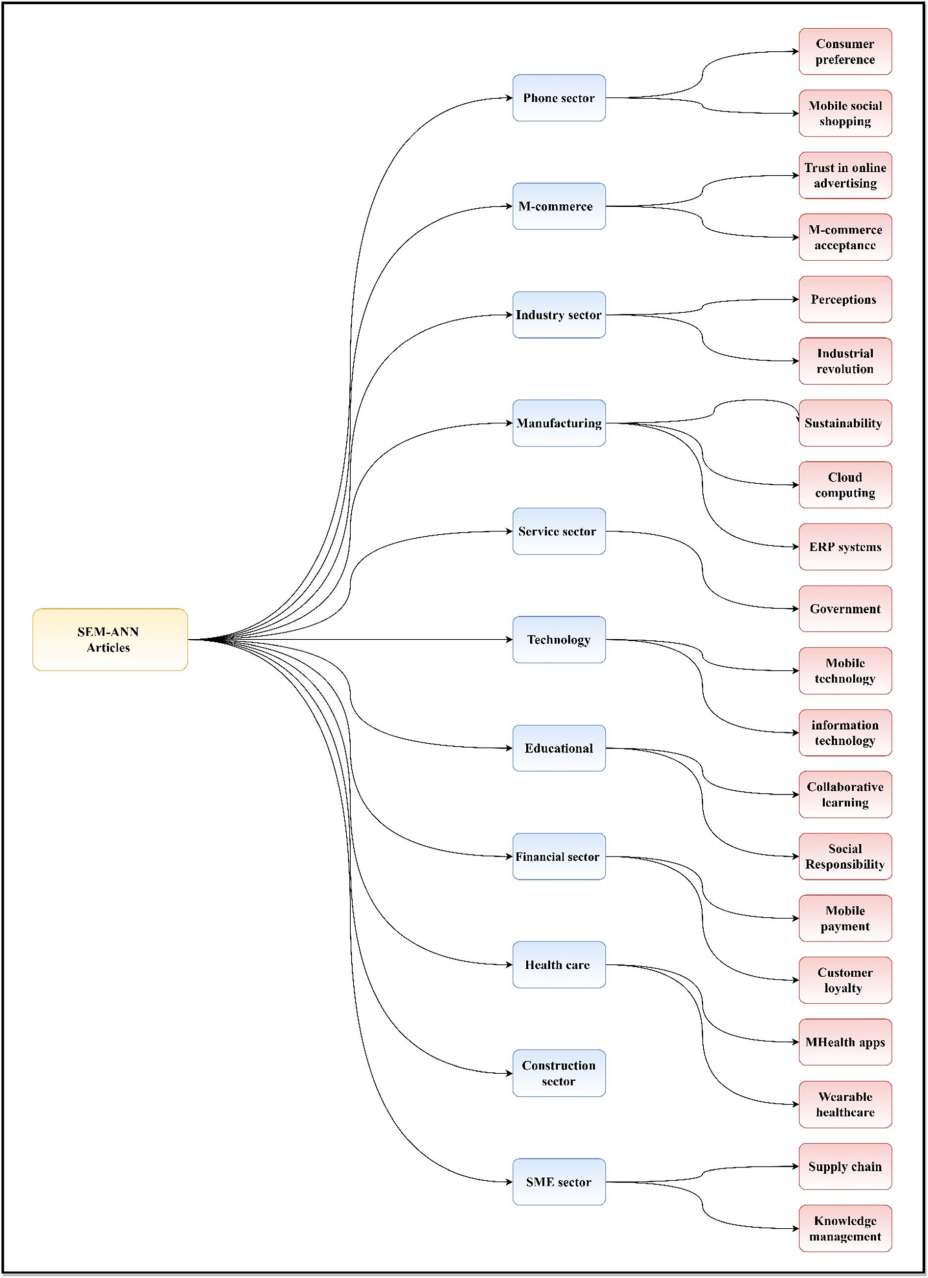
Taxonomy of literature on SEM–ANN

The articles that satisfied our criteria (shown in Fig. 1) were included in our work. Our initial search results produced 239 relevant publications, including 61 from ScienceDirect, 6 from IEEE *Xplore*^®^, 123 from Scopus, and 49 articles from WoS. A total of 12 duplicated articles were found, and thus, 227 publications were left. After scanning the titles and abstracts, 143 unrelated articles were eliminated; hence, 84 items remained. After the entire text was reviewed, 24 papers were eliminated, leaving 60 articles in the final collection. These papers were examined thoroughly and presented in this developing field with a broad map of study (Fig. 2). In addition, the collected final papers were comprehensively analysed for the theme of the present research to address all technical and scientific problems. In the literature of this research topic, various issues, such as motives, difficulties, constraints and suggested solutions, were discovered and categorised.

### SEM–ANN taxonomy

This review of SEM–ANN models provides a state-of-the-art mapping analysis and evaluates studies from different fields. In this regard, the ScienceDirect, IEEE *Xplore*^®^, and WoS databases were checked. A total of 60 papers were collected from 2016 to 2021. Several studies were conducted on the subject; however, these studies had different purposes. Accordingly, studies were classified in accordance with their purpose, and the result was used to create a taxonomy. Figure 2 illustrates the taxonomy used to review the articles focusing on SEM–ANN. This taxonomy provides a complete framework of different studies and their applications. Our classification focused on 11 major classes. The first class includes papers on the phone sector (6/60 papers). The second class is composed of papers from the mobile commerce (m-commerce) sector (6/60 papers). The third class comprises articles on the industry sector (5/60 papers). The fourth class consists of manufacturing sector articles (11/60 papers). The fifth class is composed service sector papers (2/60 papers). The sixth class includes technology sector papers (7/60 papers). The seventh class comprises educational sector articles (6/60 papers). The eighth class consists of financial sector papers (6/60 papers). The ninth class is composed of healthcare sector papers (5/60 papers). The tenth class includes papers on the construction sector (2/60 papers). The eleventh class comprises articles on small and medium-sized enterprises (SME) sector (4/60 papers). The succeeding sections describe these categories.

#### Phone sector

This category includes papers that discussed a phone sector-related aspect of SEM–ANN. This section contains six (*n* = 6) papers. The subcategory features major topics wherein SEM–ANN was adopted in (1) consumer preference and (2) mobile social shopping.

*The first set of studies* that discussed SEM–ANN with consumer preference includes three (*n* = 3) papers. Consumer demand research plays a critical role in the production of consumer goods. SEM–ANN can assist designers in determining and mapping how product characteristics influence customer preferences and in further understanding variables that influence user expectations and their inner relationships [51]. Consumers tend not to keep their smartphones for long because their preferences are constantly changing in this digital age. The authors used a two-stage approach (i.e. SEM–ANN) to elucidate the decision-making process towards smartphone buyback [18]. Another study investigated customer intention to use wearable payment [52]. *The second set of studies* that discussed SEM–ANN with mobile social shopping includes three (*n* = 3) articles. Customers may experience social network exhaustion when using mobile social platforms, in accordance with [53], which investigated mobile social marketing using mobile social tourism platforms for tourism products and services amongst local Malaysian tourists who visited George Town, one of the World Cultural Heritage sites in Penang, Malaysia. Social media exhibit high capability in managing emergencies. Furthermore, SEM and ANN were used to understand the intent to use social media during emergencies [13]. Given these reasons, interest in mobile phones is increasing in the aspects of purchase and payment. Thus, another study developed a model based on SEM–ANN to predict factors that affect decision to use mobile payment [54].

#### M-commerce sector

This category includes studies on the m-commerce sector that is related to applying SEM with ANN. This section comprises six (*n* = 6) articles. The subcategory contains major topics wherein SEM–ANN was applied to (1) trust in online advertising and (2) m-commerce acceptance.

*The first set of studies* that discussed SEM–ANN with trust in online advertising includes three (*n* = 3) articles. Trust is a concern in electronic commerce. The first study in this field explored the determinants of confidence in electronic commerce (e-commerce) in accordance with presence and social support theories by adopting a hybrid approach (i.e. SEM–ANN) that can discover nonlinear and non-compensatory relationships [55]. Similarly, trust plays a crucial role in online advertising because advertisements are affected by consumer confidence. The literature on this topic contributes to the adoption of SEM–ANN to explore factors that influence trust in online advertising [56].

Moreover, engaging customers in a virtual brand community has become a challenge to many companies. To identify factors that drive customer engagement, an SEM approach was used with ANN to recognise factors that affect the perceived ​​and social values ​​of a virtual brand community [57]. The second set of studies that discussed SEM–ANN with m-commerce acceptance includes three (*n* = 3) articles. In accordance with researchers, an essential factor that determines the extent to which any innovation can adopt is innovation resistance. In this regard, the acceptance of the mobile wallet application by customers is a challenge that is worth investigating. The authors of [58] used SEM with ANN to examine the inhibitors of mobile wallet adoption from the perspective of resistance to innovation theory. The authors of [59] aimed to identify the major factors that influence consumer adoption of m-commerce using SEM–ANN. M-commerce has expanded dramatically in recent years. Therefore, factors that predict consumer satisfaction in M-commerce have been studied in accordance with ANN and SEM [6].

#### Industry sector

This category includes studies that discussed the application of SEM–ANN in the industry sector. This section consists of five (*n* = 5) studies. The subcategory includes major topics wherein SEM–ANN was applied to (1) perceptions and (2) the Industrial Revolution.

*The first set of studies* that discussed SEM–ANN with perceptions includes three (*n* = 3) papers. The first study [8] aimed to evaluate purchasing determinants that affect customers’ perceptions of environment-friendly cars. In the context of the transportation industry, the literature has focused on studying the effect of the smart transportation systems of service providers, particularly taxi drivers who offer mobile payment for their service to passengers [60]. Finally, the effect of the qualitative aspects of online user-generated content on customers’ brand attitudes was investigated using the dataset from TripAdvisor (an online travel agency provider) [61]. *The second set of studies* that discussed SEM–ANN with the Industrial Revolution includes two (*n* = 2) articles. In [62], the authors investigated how the absorptive capability, success expectancy, commitment expectancy, firm size and top management support of cloud computing technology can contribute to the innovativeness and performance of industrial firms through adopted SEM and ANN methods to explain the next Industrial Revolution in Malaysia. Thus, the Industrial Revolution caused many changes in work, increasing victimisation in the workplace and generating social stressors with crucial implications for employee well-being. Another study examined the effects of favouritism, cynicism and gender on work withdrawal by applying SEM and ANN [63].

#### Manufacturing sector

This category includes studies that discussed the application of SEM–ANN in the manufacturing sector. This section has eleven (*n* = 11) studies. The subcategory contains major topics wherein SEM–ANN was applied to address (1) sustainability, (2) cloud computing and (3) enterprise resource planning (ERP) systems.

*The first set of studies* that discussed SEM–ANN with sustainability includes eight (*n* = 8) papers. The first study investigated the implementation of green supply chain management practices and their effects on sustainability using SEM and ANN [7]. Meanwhile, the benefit of donating food affects the sustainable management of food recovery and redistribution. Another work used SEM and ANN to explore the patterns and dynamics of food donation and distribution processes in Australian organisations [14]. Transformational operations face challenges in integrating sustainability practices into their procedures. Therefore, the authors of [64] aimed to analyse the factors involved in sustainability practices by collecting data from manufacturing companies that have adopted sustainable practices and are applying SEM–ANN. Similarly, the responsibility of a product to the environment cannot be fulfilled without the participation of customers. Thus, the latest study in the field of sustainability intended to explore the role of consumers in recycling smartphones [65]. Accordingly, the issues of sustainability and social responsibility are essential. Finally, the influence factors of eutrophication in Dongting Lake were studied [66]. In environmental management, reverse logistics is essential for sustainability. Thus, the consumer limitations of manufacturers to their reverse behaviour were investigated using SEM and ANN [67]. Adopting green information technology (IT) enhances the sustainability of manufacturing companies. Factors that affect the intention of decision makers to embrace green IT were investigated [68]. Another study aimed to examine the effects of personal and standard appeal factors on intention to adopt conservative agriculture practices and their influences on sustainable farm performance [69]. *The second set of studies* that discussed SEM–ANN with cloud computing and ERP systems includes three (*n* = 3) articles. Cloud computing has been labelled as the fifth utility, after water, gas, power and telecommunications. The first work aimed to predict the drivers of cloud computing adoption in manufacturing organisations using a hybrid two-stage SEM–ANN model [70]. The objective of [71] was to discuss the effects of cloud computing integration and external integration on the relationship between business performance and supply chain integration in the Indian context by adopting SEM–ANN. For ERP systems, SEM–ANN provides better results than the one-step SEM approach. The last study in this field aimed to identify factors that affect the acceptance and use of ERP systems in manufacturing companies [15].

#### Service sector

This category includes studies related to the application of SEM–ANN in the service sector. Only two (*n* = 2) articles belonged to this section. The first work [72] explored the effects of company size and industry type on total quality management practices in government service organisations. Similarly, mobile applications have become the preferred method of the government sector, and these applications have contributed to providing services to citizens. In [73], mobile applications for government services were studied to explore their intent to use by extending unified theory of acceptance and use of technology (UTAUT).

#### Technology sector

This category includes studies in which SEM–ANN was applied in the technology sector. This section has seven (*n* = 7) studies. The subcategory comprises major topics wherein SEM–ANN was applied to (1) mobile technology and (2) IT.

*The first set of studies* that discussed SEM–ANN with mobile technology includes three (*n* = 3) papers. In [74], a smartphone credit card was investigated because it is an emerging payment method. However, the adoption level of this method was not encouraging. Hence, factors that affect the acceptance of mobile technology were studied using PLS-SEM–ANN. In addition, the new phenomenon of government’s security response systems was explored (i.e. mobile technology) to identify important factors from the user’s perspective to create a smart government system for crisis management [75]. In addition, the critical determinants of contact tracing applications were explored using SEM and ANN [76]. *The second set of studies* that discussed SEM–ANN with IT includes (*n* = 4) articles. The first investigation related to this category aimed to identify the determinants of the technology environment model used in the study, i.e. SEM, ANN and interpretive structural modelling, to analyse factors that affect cloud computing adoption in the context of Indian organisations [77]. Similarly, IT (e.g. social media) plays an essential role during crises, such as the COVID-19 pandemic. Consequently, how motivational factors and personality traits affected the sharing of unverified information during the COVID-19 pandemic was investigated [9]. In the current globalising world, IT has become critical for companies and an inseparable part of social and economic life. Consequently, the use of IT on the performance of supply chain operations was analysed [78]. However, the adoption of IT applications by consumers faces many obstacles that require in-depth analysis. Barriers to the adoption of electronic and smart metres by consumers were studied using UTAUT2 [79].

#### Educational sector

This category includes studies regarding the application of SEM–ANN in the educational sector. This section is composed of six (*n* = 6) papers. The subcategory includes major topics wherein SEM–ANN was applied to (1) collaborative learning and (2) social responsibility.

*The first set of studies* that discussed SEM–ANN with collaborative learning includes three (*n* = 3) papers. The socially cooperative approach to education exerts an important influence on the cognitive assimilation of learning in a student’s life. Many social media, including Facebook, have made considerable achievements in the academic field. In the first and second articles, determinants that limit students’ intent towards the academic use of Facebook were explored [80, 81]. In addition, students’ motivations for adopting mobile learning and determinants that affect behavioural intention to adopt mobile learning were explored using SEM and ANN [82]. *The second set of studies* that discussed SEM–ANN with social responsibility includes three (*n* = 3) articles. The first work in social responsibility was concerned with measuring the contribution of human resources and its effect on administrative efficiency in higher education institutions [83]. Social responsibility is essential for the competitiveness of organisations. Therefore, the effects of the social responsibility factors of organisations on a university’s competitiveness towards other universities were investigated using SEM and ANN [16]. From another point of view, university students were selected as subjects to determine the effects of emotional eating, body shape concerns and measures of body appreciation on preventive behaviour [84].

#### Financial sector

This category includes studies on SEM–ANN application in the financial sector. The section comprises six (*n* = 6) articles. The subcategory includes major topics wherein SEM–ANN was applied to mobile payment and customer loyalty.

The category that discussed SEM–ANN with mobile payment and customer loyalty includes six (*n* = 6) papers. Studying user intention to notify mobile payment is an essential issue because of facilities that offer mobile payment to users and banks. The key concept precedents of customer intent to use mobile payment were studied using SEM–ANN [85]. In terms of financial behaviour, the effects of financial anxiety, optimism, financial security, consultative thinking, attention to financial issues and preventive savings needs on the business activities of financial institutions during the COVID-19 pandemic were measured [12]. Mobile banking (m-banking) has grown rapidly because of customers’ increased use of mobile payment. A new test paradigm for understanding users’ intention in m-banking was suggested [86, 87]. Finally, customer loyalty is constantly affected due to major developments in banking services. Moreover, factors that affect customer loyalty in the banking industry of Brazil were studied [88]. With regard to financial issues, UTAUT2 was used with trust and personnel innovativeness to measure intention to adopt cryptocurrency [89].

#### Healthcare sector

This category includes studies that applied SEM–ANN in the healthcare sector. This section is composed of five (*n* = 5) articles. The subcategory includes major topics wherein SEM–ANN was applied to mobile health (mHealth) apps and wearable healthcare devices.

UTAUT2 was used in [64–66]. Hence, the adoption of mHealth apps was analysed by examining the factors that influence the behavioural intention of mHealth apps amongst the younger generation. Similarly, wearable healthcare devices improve access to healthcare information and self-managed healthcare. Despite the benefits of using wearable healthcare devices, their acceptance amongst the elderly is low. Therefore, UTAUT2 was used with additional constructs to identify important determinants that affect the adoption of wearable healthcare devices [90]. IT has been used extensively in healthcare. Nevertheless, wearable healthcare devices suffer from low acceptability amongst individuals. Consequently, the key factors that affect an individual’s intent to use wearable healthcare devices were studied [91].

##### Construction sector

This category includes studies wherein SEM–ANN was applied in the construction sector. The section contains two (*n* = 2) articles.

The first work on road safety issues was concerned with transportation policy. Factors that affect road safety policies were studied by adopting SEM and ANN due to an increase in road accidents in Spain [92]. Meanwhile, a recent research on energy systems highlighted the understanding of the behaviour of a home investor in a photovoltaic power system [93].

##### SME sector

This category includes studies in which SEM–ANN was adopted in the SME sector. The section comprises four (*n* = 4) articles. The subcategory includes major topics in which SEM–ANN was applied to (1) the supply chain and (2) knowledge management.

*The first set of studies* that discussed SEM–ANN with supply chain includes two (*n* = 2) papers. In [94], the authors investigated the effects of complexity, comparative advantage, cost, top management support, market dynamics, organizational support and competitive pressure on the blockchain adoption of operations and supply chain management amongst SMEs. In addition, a customer relationship strategy adopted by SMEs was investigated [95]. *The second set of studies* that discussed SEM–ANN with knowledge management includes two (*n* = 2) articles. The first study examined knowledge management practices, technological innovation and competitive advantage in SMEs [20]. SMEs are affected by many factors, including cloud computing. Thus, the effect of cloud computing on SME performance was investigated by combining SEM with ANN [96].

### Comprehensive science mapping analysis

The application of bibliometrics is increasingly expanding across various fields. This technique is particularly suited for scientific mapping during a period when emphasis on empirical input has resulted in numerous, fragmented and contentious research streams [97]. As mentioned previously, the articles in this review can be divided into the following 11 categories: phone, m-commerce, industry, manufacturing, service, technology, education, financial, healthcare, construction and SME sectors. The total numbers of published articles selected in this review are as follows: ScienceDirect (31 papers), WoS (24 papers), IEEE *Xplore*^®^ (1 paper) and Scopus (4 papers).

**Fig. 3:**
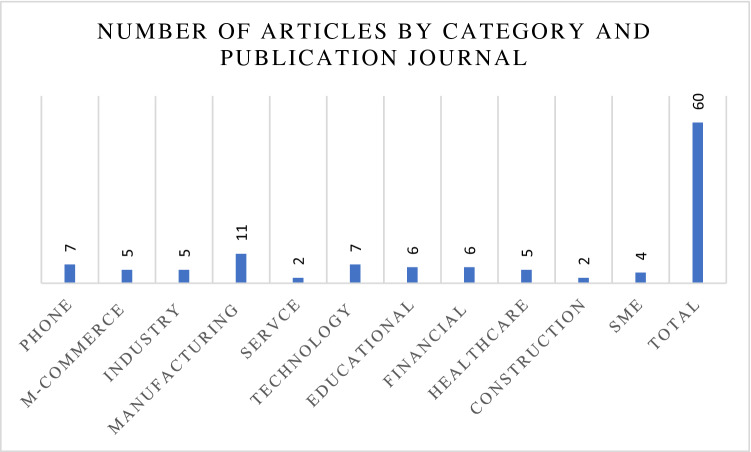
Number of articles by category and publication journal

Figure 3 shows that the percentage of articles by sector category and publication journal ranges from 3 to 18%. A comprehensive science mapping analysis was adopted to explore the problems of combining SEM with ANN in most of the cases discussed because only the advantages of combining SEM with ANN were presented. Many barriers were disregarded, and mapping analysis based on bibliometrics was used. The investigated literature listed in Table 1 can be clarified.

**Table 1:** Primary information regarding the collection

Description	Results
Primary information about data	
Time span	2016–2021
Sources (e.g. journals and books)	37
Documents	60
Average years from publication	2
References	5330
Document types	
Article	59
Conference paper	1
Keywords plus (ID)	482
Author’s keywords (DE)	229
Authors	
Authors	162
Author Appearance	226
Authors of single-authored documents	2
Authors of multi-authored documents	160

Table 1 indicates the considerable growth in the number of studies that incorporate SEM–ANN, particularly in the last 4 years, which was adopted as a criterion for selection because the number of papers was 60 for different cases and exhibited an annual growth rate of 14.87%, with 5330 references. In terms of number of authors, most of the papers were joint research with multiple authors, except for one paper with a single author.

#### Most relevant sources

**Fig. 4:**
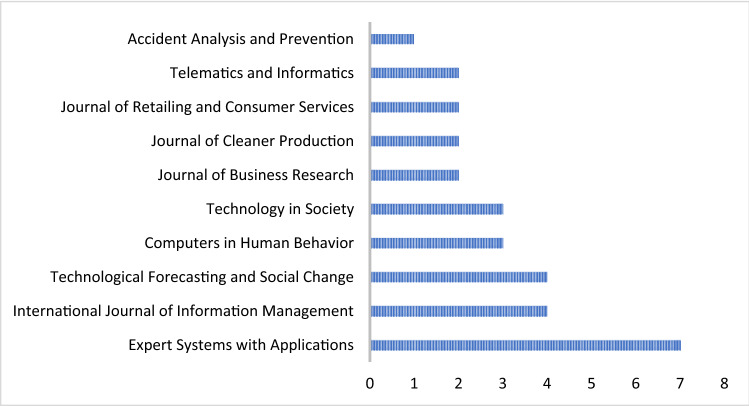
Most relevant sources

Figure 4 provides the most relevant sources for 37 journals regarding this topic. The journals that have repeatedly published papers on this subject were identified to show that these journals have been interested in SEM–ANN recently. We noted that the journal ‘Expert Systems with Applications’ published five papers. This finding is expected because it specialises in this field.

The percentage of the final set of articles ranges from 7 to 12% in terms of the most relevant sources. Moreover, the journal ‘Expert Systems with Applications’ published a high number of articles (i.e. 7% of the papers), followed by the International Journal of information Management.

#### Country-specific production

**Fig. 5:**
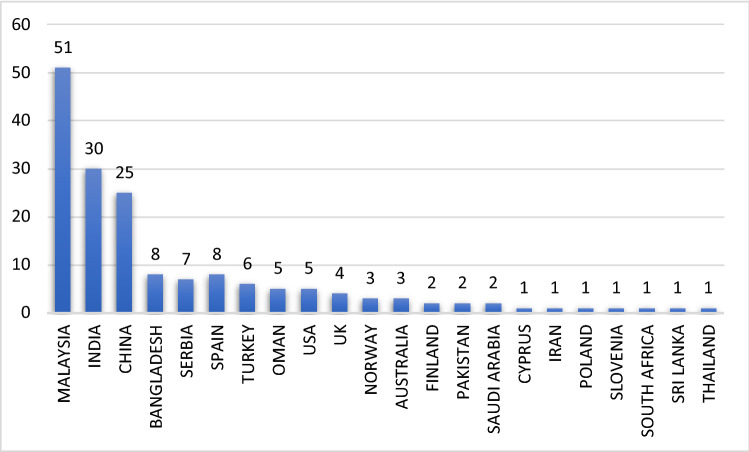
Country-specific production

Figure 5 shows that the articles on SEM–ANN included in this review came from 168 countries. The articles generally include case studies conducted in these countries.

As indicated in the table, the countries that produced the most number of papers on the topic of SEM–ANN are Malaysia, India and China. In terms of country-specific production, the highest percentage was achieved by Malaysia (30%), followed by India (18%) and then China (15%).

#### Annual production

**Fig. 6:**
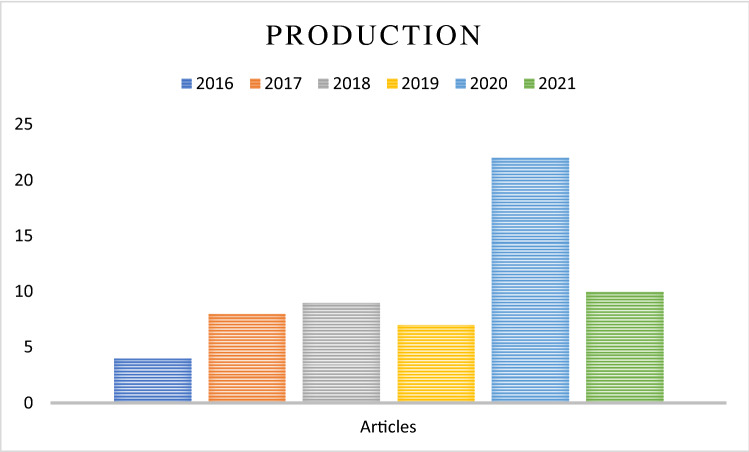
Annual production

Figure 6 illustrates the relationship between annual production and year of publication. The trend depicts a dramatic increase in interest in 2020.

In accordance with year of production, the percentage of reviewed papers ranges from 7 to 37%. Moreover, the annual production in 2020 represents the largest number of papers during the study period. Most of these papers are technology cases, and the rise in their number corresponds to numerous problems associated with this topic in recent years. Technology cases are followed by marketing cases and topics on customer perception. Most cases discussed a specific matter or identified indicators or factors and their order in accordance with importance.

#### Word cloud

**Fig. 7:**
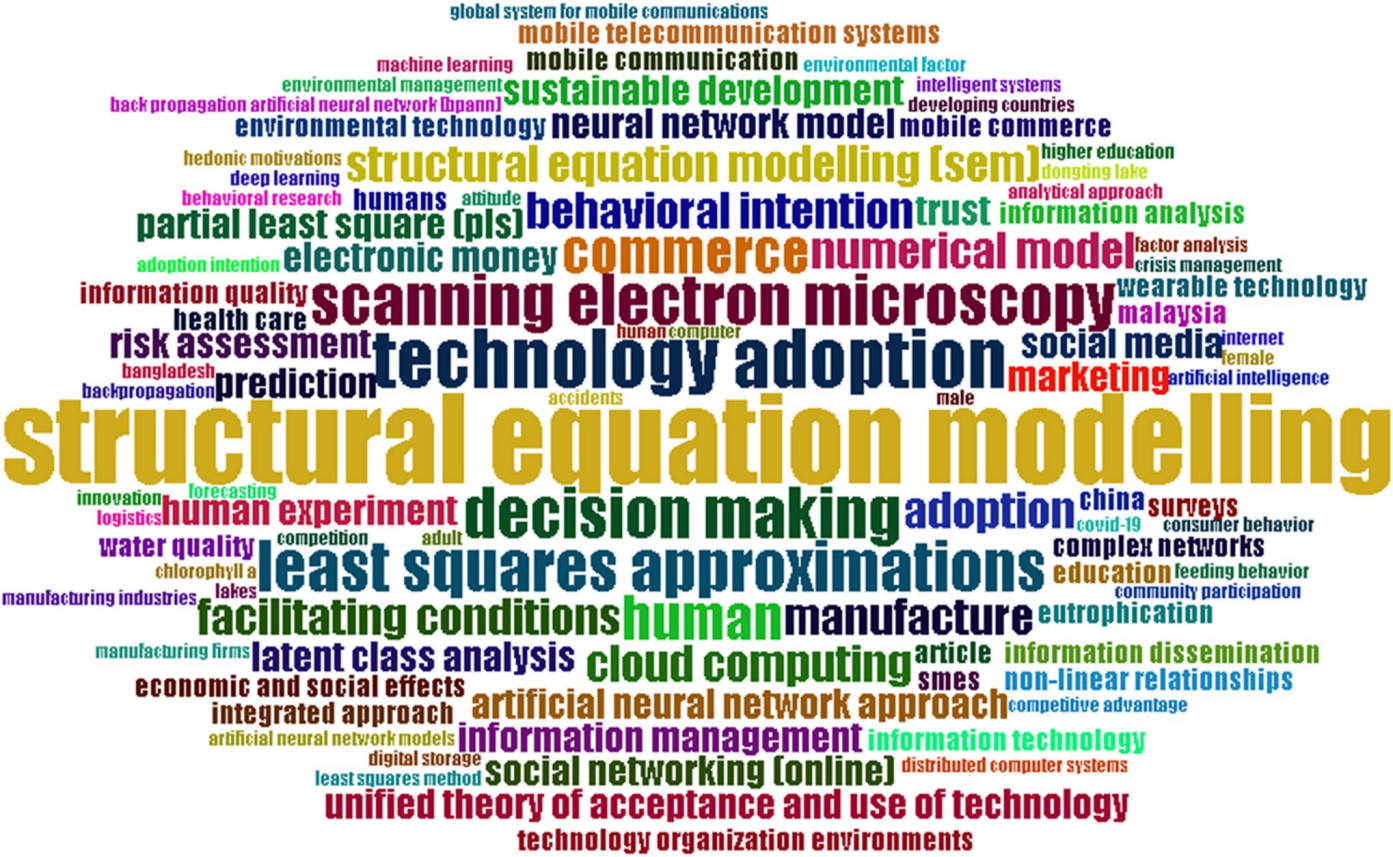
Most frequent words

A word cloud refers to the most important topics that deal with a certain subject. Figure 7 presents the essential critical words adopted by previous studies on SEM–ANN and the relationships amongst them.

A word cloud can provide support to the research taxonomy by dividing SEM–ANN studies into four categories. As shown in Fig. 7, most papers focused on the technological aspects (i.e. mobile payment, m-commerce, health apps, sustainability and green issues, trust in social media, UTAUT and cloud computing. The keywords indicate that the authors who investigated SEM with ANN were also interested in these areas.

## Discussion

The four key facets of the current review’s scholarly literature analysis of SEM and ANN studies are discussed in the succeeding subsections. These considerations are the impetus for adopting various approaches and methods that can describe the causal interaction between input and output and offer a new perspective for developing nonlinear models. Comprehensive science mapping analysis can be conducted to explore new research directions related to SEM–ANN integration. Lastly, existing challenges in SEM and ANN studies and the recommended solutions to overcome these challenges are presented.

### Motivations

**Fig. 8:**
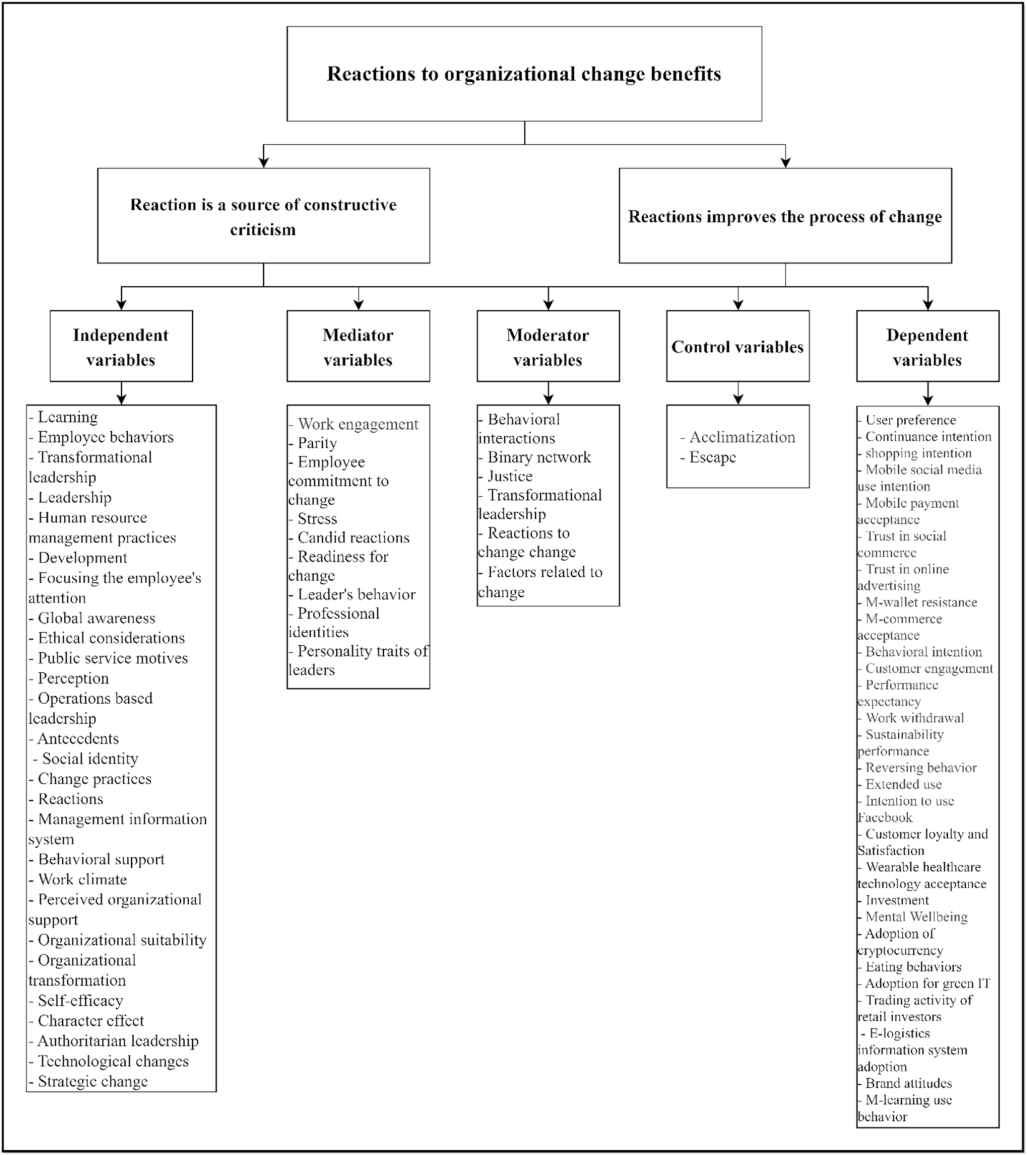
Categories of the benefits of combining SEM with ANN

The benefits of using SEM combined with ANN are evident and compelling. This section identifies the benefits mentioned in the literature organised into groups on the basis of similarities. For further discussion, the corresponding sources are provided (Fig. 8).

As shown in Fig. 8, the use of the multi-analytical two-phase SEM–ANN method has two major benefits. First, this approach allows further validation of SEM analysis findings. Second, it captures linear and dynamic nonlinear interactions between antecedents and dependent variables and provides a more accurate measure of each predictor’s relative power.

#### Explaining causal relationship

SEM can determine relationships amongst unobserved constructs (i.e. independent, mediator, moderator, control and dependent variables) [53]. This research field is distinguished by examining various causal relationships amongst variables. Major interests include behavioural intention and new technology acceptance (e.g. mobile payment, m-commerce, health apps, sustainability and green issues, trust in social media, UTAUT and cloud computing) [7, 53–55, 59, 60, 71, 98]. The combination of SEM and ANN in this type of research involves the adoption of technology, concern for resistance to innovation and enhancing perceived responsiveness [58]. Moreover, the use of deep learning-based dual-stage PLS-SEM and ANN analysis provides more in-depth test findings than a single-step PLS-SEM, allowing for the identification and prediction of variables [89]. PLS-SEM outperforms CB-SEM in terms of the statistical precision of causal theories [87]. PLS-SEM is becoming a common and widely used tool in social science. Many researchers have used PLS-SEM to solve problems that cannot be addressed by CB-SEM [90].

Furthermore, this method is a good technique for designing and evaluating theories and analysing the interactions amongst variables; conceptual constructs include independent, mediating, moderating and dependent variables [20]. Hence, PLS-SEM is appropriate for explaining causal relationship. In addition, PLS-SEM exhibits predictive advantages, such as *R*^2^ and *Q*^2^ values [51]. PLS-SEM deals with an elaborate conceptual framework that consists of moderating and mediating models; thus, it is in line with the previous literature that deals with SEM in terms of understanding and predicting the determinants of constraints [14]. In conclusion, SEM detects linear and compensatory relationships amongst constraints to explain the effect mechanism amongst them [61, 82].

#### Providing a nonlinear model

As mentioned in the previous section, one benefit of SEM is its capability to explain linear and compensatory causal relationships amongst constraints. However, SEM cannot explore nonlinear relationships amongst constructs [16]. Hence, ANN analysis has become an essential method that complements PLS-SEM in recent research [88]. ANN can detect nonlinear and non-compensatory relationships by relying on the compensatory model, enabling it to address complex processes in human decision-making [90]. Moreover, ANN provides the rank and validates the significant predictors of a model [20]. In addition, ANN deals with linear and nonlinear associations because it depends on the black box process of the algorithm, which is the basis for prediction [99]. Thus, the multilayer expectations of a feedforward backpropagation (BP) ANN algorithm have generated normalised importance [89]. In accordance with [18, 55, 56, 62, 70], the benefits of combining SEM with ANN are consumer preference analysis, smartphone repurchase, illustrating the mechanism of trust in online advertising and predicting the motivators that affect cloud computing adoption. In addition, ANN validates SEM results and understands the most important aspects [84]. However, ANN analysis is unsuitable for testing hypotheses because it depends on the black box process of the algorithm [6]. To capture linear, testing hypothesis and nonlinear relationships amongst constructs, SEM and ANN analyses were combined to overcome the weaknesses of SEM [71]. Meanwhile, ANN helps assess the relative importance of the determinants of mobile app adoption in various industries [6, 13, 53, 54, 58–60, 73, 74, 82, 85, 87, 99]. In summary, using ANN with SEM provides a new perspective on building nonlinear models [7, 65].

### Challenges and limitations

**Fig. 9:**
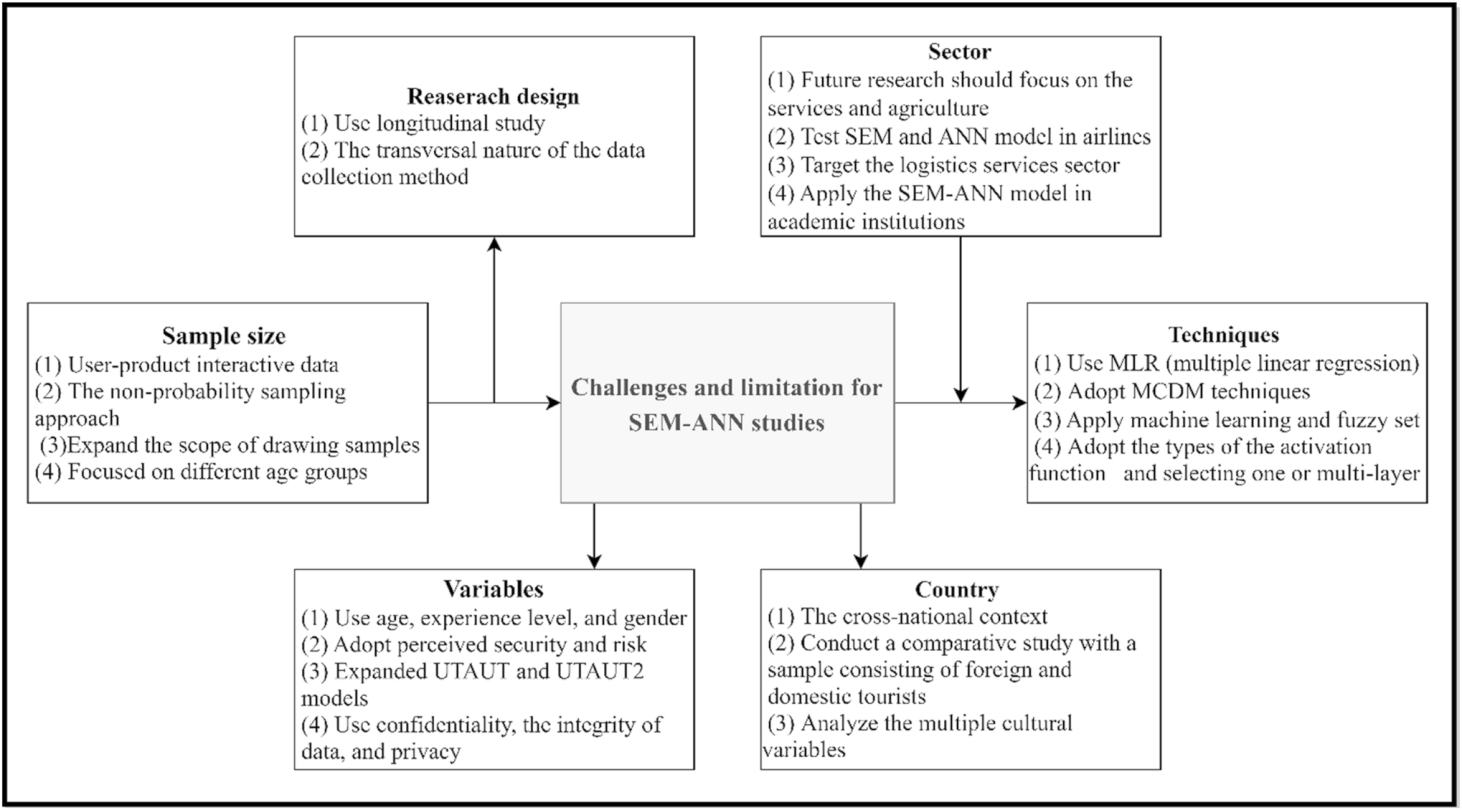
Challenges and limitations of SEM–ANN studies

The combined SEM and ANN approach exhibits several problems. Several challenges in SEM–ANN are described in the literature. Research design, sector, country, research sample, techniques and variables are amongst the challenges. This section focuses on reviewing the challenges presented in Fig. 9.

#### Research design

Research design refers to the general strategy selected by the researcher to integrate various components of a study coherently and logically. Shifting study planning into longitudinal study is also a pattern for prospective researchers because observing how market expectations continue over time is interesting [13, 18, 20, 73, 74, 79]. Furthermore, the transversal nature of a data collection method hinders the appropriate assessment of users’ behavioural development. Therefore, the longitudinal approach is appropriate because it can test the strength of relationships [7, 54–56, 58, 62, 85, 98].

#### Sector

In accordance with the research objectives, a sector represents the choice of applying the study to public or private organisations. Most of the reviewed literature focused on mobile payment issues, m-commerce, health apps, sustainability and green issues, trust in social media, UTAUT and cloud computing. However, the sample does not allow generalisation regarding the results. Therefore, the new technology sector represents a challenge and constraint for future research to focus on other sectors [8]. Furthermore, the findings are mostly obtained from industrial firms. Different sectors and geographical locations can produce varying outcomes [62]. Consequently, making a generalisation to all manufacturing companies is the primary concern. Future research should replicate the proposed model in other sectors, such as services, construction, materials, agriculture and vehicles in different regional areas and categories of the manufacturing sector [59].

However, other authors are interested in the hotel industry, which influences generalisation, and future studies can apply the proposed model to different sectors, such as airlines, restaurants and hotels [63, 74]. In terms of sustainability issues, the effect of green practices on the sustainability performance of manufacturing companies has been investigated, and the results of such studies cannot be generalised across all industries. Moreover, authors can focus on companies that provide transportation and logistics services to further investigate green challenges and carbon dioxide emissions caused by the transportation industry [7, 14]. Other studies have applied SEM and ANN to selected public universities to collect data by conducting surveys, leading to bias. A study with a larger sample size can be conducted in other academic institutions to avoid this issue [80]. The studies reviewed here were restricted to SMEs. Future work may narrow down the sample size to downstream industries, such as food and hardware products [20].

#### Country

Most of the reviewed studies focused on one country without considering the cultural differences between countries, leading to difficulties in applying the results to other countries. Consequently, researchers are advised to focus on other nations, particularly developing countries, to determine whether the moderate results and gender norms will continue. Future researchers should consider comparing two or more nations [18, 74, 79]. Other authors have chosen local tourists, and academics can conduct a comparative study with samples consisting of foreign and domestic tourists in a multinational context [13, 53, 55, 87, 98]. Moreover, the assessment of intent to use and acceptance of new technology can be used as a basis for investigating other countries and comparing the results with Spain to study differences between cultures and identify the determinants of acceptance for the same technology [54, 80, 98]. As mentioned in Sect. 4.2, most of the studies focused on the Malaysian context. The results were limited to the Malaysian perspective, and thus, a new study should be conducted in other geographical areas [56, 59, 63]. Most of the studies were performed in Asian countries, and therefore, the results are unsuitable for generalisation; such research should also be conducted in European countries [58, 71, 85]. This region poses a challenge to the literature because it is linked to cultural and economic contexts. That is, analysing multiple cultural variables that influence consumer behaviour will be interesting [8, 64, 70, 74, 77, 95].

#### Research sample

The primary objective of determining sample size is obtaining data, whether primary or secondary, to finalise the results that contribute to practical and theoretical implications for practitioners and academics. Most of the studies focused on collecting data from users to explore and analyse consumer preference. However, further studies still exhibit potential because user–product interactive data can be collected and used to improve the accuracy of models [51, 71]. Moreover, the selected samples affect the generalisability of the results. Therefore, researchers are encouraged to expand their scope in selecting samples to enhance the representation of sample size [18]. The other issue is the non-generalisability of probability sampling approaches. Future research should suggest a non-probability sampling approach [53]. One study used countries belonging to the Gulf Cooperation Council as sample size and focused on age groups ranging from 20 to 40 years. In conclusion, research may explore different age groups to see potential differences in mobile technology acceptance [73, 74, 98]. In addition, the snowball sampling technique for data collection can be avoided and researchers can focus on cluster sampling in SEM and ANN studies [80]. One study collected data from users via Facebook; however, other studies can be conducted by targeting social platforms, such as WhatsApp and Telegram [81]. Many empirical studies highlighted the adoption of social responsibility on the basis of the response of owners of SMEs. Further adopting the perspectives of SME owners/managers and employees may reduce sample size bias [95, 96].

#### Techniques

Several authors have used a quantitative research method (e.g. 100% self-administered survey), and many nonverbal communication data obtained through qualitative or mixed research methods have not been considered. Qualitative research methods, such as observations and in-depth interviews, can overcome some of the information deficiencies of quantitative methods [7]. Hypotheses are tested with a survey questionnaire when applying SEM–ANN. Thus, the literature on this issue has focused on survey studies. However, authors can use other statistical and analytical methods, such as multiple linear regression (MLR), analytical network process, decision-making trial and evaluation laboratory and the Technique for Order of Preference by Similarity to Ideal Solution [70, 71, 74, 77]. ANN adopts a BP learning methodology that some authors have used. Radial basis function (RBF)-learning ANNs can be developed during the early stages of research wherein neural networks trained in BP outperform ANN networks trained in RBF. Other training methods are available, such as Bayesian-trained ANNs, or unsupervised learning methods, which may be examined and used in subsequent studies [72]. A more comprehensive statistical analysis is critically needed to identify interesting results, particularly a qualitative analysis that helps discover additional information and implications for practitioners and academics [98]. The authors validated the study model using SEM and ANN without considering a multi-staged computational approach that adopts other nonlinear and non-compensatory methods, such as random forest, support vector machine and fuzzy set, which exhibit robust predictive capabilities [90]. The results differ under a multiple set of deep ANN architecture, and a comparison can be made amongst the results of previous studies to determine the best architecture for their scenarios [52]. Deep ANN requires researchers to equate ANN findings with one and more than one layer to achieve consensus on the right architecture [89]. Thus, ANN software has three activation functions: identity, hyperbolic tangent and sigmoid). Several other widely used and well-known activation functions are available; thus, adopting and comparing a framework with those activation functions are crucial [6]. Moreover, other neural networks, such as machine learning and genetic algorithms, should be used [84].

#### Variables

The variable referred to in the literature review was asserted as the effect of perceived ease of use on mobile shopping intention because of ample experience. Thus, authors can select inexperienced respondents to confirm the results. Some scholars have investigated the connection between social media and disasters. This issue poses critical and perplexing concerns that should be further investigated: why do consumers of mobile social media exchange details during emergencies? How will the information-sharing activity of mobile social media users be improved [13]? Knowledge has been gained regarding the adoption of mobile payment, but including other variables, such as the age, experience level and gender of respondents, with regard to similar payment systems is interesting [8, 54, 73, 86]. Consequently, studies can examine gender differences in trust in e-commerce. Social network theory, social bond theory, the likelihood of detail model and the Big Five model for increasing predictive power can be adopted [55]. ANN cannot explain 100% confidence in online advertising because it focuses on specific variables. More theories increase the predictive power of a research model [56]. Similarly, ANN can predict with an accuracy of 76.4%. Future studies may conduct a comparative analysis of consumer resistance to mobile wallet between industrialised and emerging economic contexts [58]. However, factors such as perceived security and perceived risk can be used to affect electronic transactions [59, 64]. Many studies have adopted UTAUT and UTAUT2. In addition, several models for technology acceptance focus on information systems. Research models can be combined or expanded to enrich results and provide a comprehensive view of factors, such as cloud computing [62]. The literature has suggested several determinants that affect consumer participation in environmental management, such as environmental concerns, assignment of responsibility and knowledge [65]. Hence, the literature review should extend the current theoretical model to provide a new perspective for building nonlinear models [74]. Previous studies have analysed the effect of a limited number of precedents on intention to use technology. Adopting other factors, such as comparative advantage, perceived awareness, suitability, experiential motivation, familiarity with technology and personality traits, will be crucial [85]. Hence, the literature must test the effects of confidentiality, integrity of data and privacy on adoption decisions and the role of a blockchain in protecting sensitive information [94].

### Recommendations

**Fig. 10:**
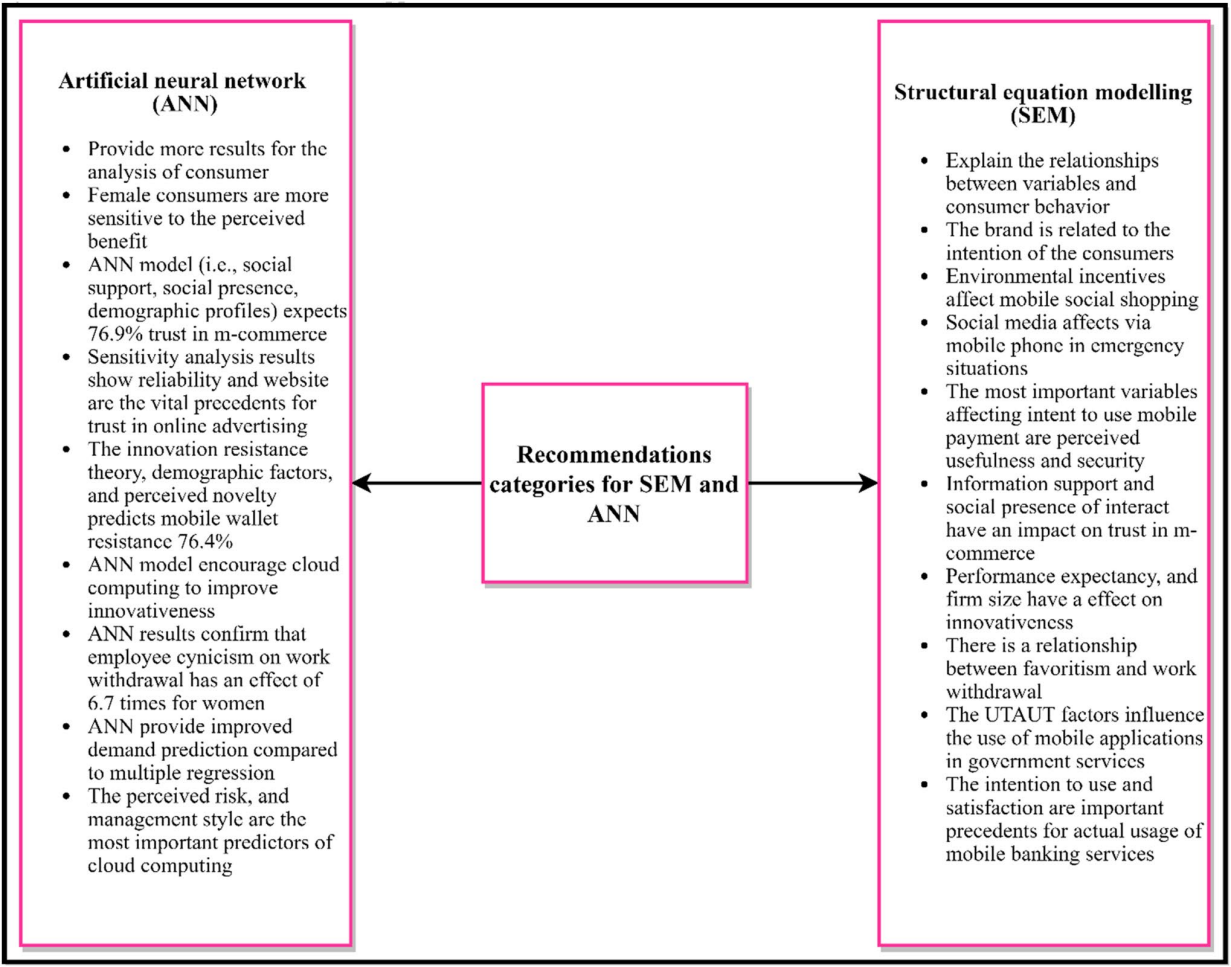
Recommendation categories for SEM–ANN studies

As illustrated in Fig. 10, this study presents recommendations from the literature to address the issues experienced by diverse areas in SEM and ANN approaches. The recommendations are classified on the basis of their nature.

#### SEM recommendations

SEM was introduced to explain causal relationships amongst variables and analyse consumer behaviour to understand factors that influence user perceptions of a smartphone [51]. Brand is related to the intention of consumers to buy back smartphones through the intention of continuity [18]. SEM has demonstrated that environmental incentives (i.e. social presence, perceived mobility and quality of service) affect mobile social tourism through perceived usefulness and enjoyment [53]. Social media affect via mobile phones during emergencies [9, 13]. SEM was used to determine factors that exert an effect on mobile payment adoption. The authors concluded that the most important variables that affect intent to use are perceived usefulness and perceived security [54, 74, 85]. In addition, information support and social presence of interact with vendors and income exert an effect on trust in m-commerce [55, 59]. Education, risk barrier, usage barrier, tradition barrier, value barrier, income and perceived novelty substantially affect mobile wallet resistance [58].

By contrast, government financial aid and reliability are the major drivers for electric vehicles. In addition, limited range, shipping time and reduced infrastructure availability negatively affect perceived reliability [8]. The results of other authors indicate that performance expectancy, absorptive capacity and firm size positively affect innovativeness [62]. On the organisational side, favouritism influences employee withdrawal and ridicule from work, and underestimating an employee has a positive effect on work withdrawal and mediates the relationship between favouritism and work withdrawal [63]. The recommendations of the authors outline the importance of green supply chain management practices as strategies for improving sustainability [7, 64, 68]. The SEM results showed that risk analysis, IT security risks, management style, technology innovation and trust affect the use of cloud computing [70, 96]. SEM has demonstrated that various factors (e.g. data quality, system complexity, system performance, user manual, social influence, business process fit, training and education, support, communication, ERP work compatibility, ERP usefulness, ERP ease of use and attitude towards ERP systems) exert a dynamic effect on the use of ERP systems [15]. SEM has been used in other studies to determine dynamic parameters (i.e. UTAUT factors, attitude to use, information quality, means of uncertainty reduction and channel characteristics) that influence the use of mobile applications in government services [73, 75]. The results inferred from SEM show that perceived benefit, resource sharing, social cooperation, perceived enjoyment and UTAUT2 factors are the most influential determinants of Facebook use for educational issues, mHealth apps, and cryptocurrency adoption [76, 80–82, 89, 98]. In addition, social responsibility exerts an effect on increasing the competitiveness of universities [16]. By contrast, intention to use and satisfaction are important precedents for the actual usage of mobile banking services [86]. Finally, performance expectancy, social influence, self-actualisation, functional unity and hedonic motivation affect wearable healthcare technology and payment acceptance [52, 90, 91].

#### ANN recommendations

ANN can take advantage of the nonlinear nature and provide more reliable results for analysing consumer preferences [51]. Moreover, female consumers are more sensitive to perceived benefits, whilst male consumers are more sensitive to brand [18]. ANN results explain the importance of intent to use social media during emergencies [9, 13]. The results of a neural network analysis confirm the findings of SEM by providing a different order of influence of important adopters of mobile payment [54]. The ANN model (i.e. social support; social presence; demographic profiles of gender, age, education and income) expects 76.9% trust in m-commerce [55, 59]. Sensitivity analysis results in accordance with ANN show that reliability, followed by website quality, reputation and hours spent, are the most crucial precedents for trust in online advertising [56]. Moreover, the ANN model (i.e. innovation resistance theory, demographic factors and perceived novelty) correctly predicts mobile wallet resistance (76.4%) [58].

In addition, discrepancies occur in the ranks of SEM and ANN predictors [8]. The predictions of ANN encourage cloud computing to improve innovativeness and firm performance [62]. ANN results confirm that the effect of employee cynicism on work withdrawal is 6.7 times higher for women. The effect of favouritism on work withdrawal is 2.1 times that of men [63]. Hence, neural networks provide improved demand prediction compared with that of MLR [14]. ANN shows that perceived risk, confidence and management style are the most important predictors of cloud computing [70, 77, 96]. Other authors have utilised the neural network model to confirm SEM results. The findings indicate that confidence and expected performance are the most effective factors of mobile applications in government services [73]. Moreover, perceived compatibility, awareness and perceived response time are crucial factors that predict adoption behaviour [75]. Similarly, privacy concerns exert a negative effect on the application of smart metre technology. Simultaneously, environmental reactions and technological awareness are critical factors that affect smart metres [79]. The results of ANN show that collaboration is the most important indicator of Facebook’s adoption for academic purposes and mHealth apps, followed by resource sharing, perceived enjoyment and perceived benefits [80, 81, 98, 99]. In accordance with ANN findings, community-oriented social responsibility is a crucial factor for increasing the competitiveness of universities [16].

## Potential future work

This study contributes two points for potential future work that are in line with the coherent comprehensive taxonomy (Fig. 2) that describes 11 sectors. To our knowledge, a new research direction regarding the most important sector (i.e. healthcare) must be investigated due to its significance when applying SEM–ANN approaches.

- First, as mentioned in the review and bibliometrics sections, most relevant sources adopted four types of cases, namely, organisation study, marketing, technology and sustainability cases. However, no study has used SEM–ANN in the autism spectrum aspect. Autism spectrum studies have confirmed that play is an important tool for parent–child connection [100]. Therefore, future work can investigate the importance of the determinants of play, musical and art therapy adoption amongst autistic children.
- Second, most of the literature is interested in exploring causal and nonlinear relationships using SEM–ANN amongst the determinants of e-commerce, mobile shopping and trust in e-commerce. However, no study has explored a crucial determinant related to the health aspect (e.g. sensing-enhanced therapy adoption). Hence, sensing-enhanced therapy regimens for evaluating children with autism spectrum disorders exhibit independent interaction capabilities and require few human resources [101, 102]. Moreover, potential future work can use SEM–ANN to determine the barriers to adopting sensing-enhanced therapy amongst autistic children.

## Conclusion

This work proposed a framework for a comprehensive literature review. The framework represented the study’s environment. A systematic study of SEM and ANN approaches was introduced to identify key challenges and limitations in implementing dual-stage SEM–ANN research. We thoroughly examined several studies to demonstrate the benefits, challenges and recommendations of using hybrid SEM–ANN, and we identified several gaps. This research provides a wider base to industry trends by analysing 11 sectors in SEM–ANN studies to gain deeper insights into the investigated area. The objective is to encourage academics and practitioners to use the multi-analytical two-phase SEM–ANN to validate SEM findings and capture dynamic nonlinear interactions between antecedents and dependent variables. Hence, a more accurate measure of each predictor’s relative power is obtained. The manufacturing and technology sectors have the highest number of investigations, whereas the construction and SME sectors have the least. This study will be a helpful reference for academics and practitioners in providing guidance and insightful knowledge to future studies (e.g. no study has yet presented an integrated solution for autism syndrome based on multi-criteria decision-making techniques with SEM–ANN). This research also resolves the ambiguity of SEM and ANN trends.
